# Network Hyperexcitability in Early-Stage Alzheimer’s Disease: Evaluation of Functional Connectivity Biomarkers in a Computational Disease Model

**DOI:** 10.3233/JAD-230825

**Published:** 2024-06-11

**Authors:** Cornelis Jan Stam, Willem de Haan

**Affiliations:** a Department of Neurology, Clinical Neurophysiology and MEG Center, Amsterdam Neuroscience, Vrije Universiteit Amsterdam, Amsterdam University Medical Center (Amsterdam UMC), Amsterdam, The Netherlands; b Alzheimer Center Amsterdam, Neurology, Vrije Universiteit Amsterdam, Amsterdam University Medical Center (Amsterdam UMC), Amsterdam, The Netherlands

**Keywords:** Activity dependent degeneration, Alzheimer’s disease, biomarkers, computational model, functional connectivity, network hyperexcitability, whole brain

## Abstract

**Background:**

There is increasing evidence from animal and clinical studies that network hyperexcitability (NH) may be an important pathophysiological process and potential target for treatment in early Alzheimer’s disease (AD). Measures of functional connectivity (FC) have been proposed as promising biomarkers for NH, but it is unknown which measure has the highest sensitivity for early-stage changes in the excitation/inhibition balance.

**Objective:**

We aim to test the performance of different FC measures in detecting NH at the earliest stage using a computational approach.

**Methods:**

We use a whole brain computational model of activity dependent degeneration to simulate progressive AD pathology and NH. We investigate if and at what stage four measures of FC (amplitude envelope correlation corrected [AECc], phase lag index [PLI], joint permutation entropy [JPE] and a new measure: phase lag time [PLT]) can detect early-stage AD pathophysiology.

**Results:**

The activity dependent degeneration model replicates spectral changes in line with clinical data and demonstrates increasing NH. Compared to relative theta power as a gold standard the AECc and PLI are shown to be less sensitive in detecting early-stage NH and AD-related neurophysiological abnormalities, while the JPE and the PLT show more sensitivity with excellent test characteristics.

**Conclusions:**

Novel FC measures, which are better in detecting rapid fluctuations in neural activity and connectivity, may be superior to well-known measures such as the AECc and PLI in detecting early phase neurophysiological abnormalities and in particular NH in AD. These markers could improve early diagnosis and treatment target identification.

## INTRODUCTION

There is increasing evidence that a disturbance in the balance between excitation and inhibition (E/I) in neural circuits plays an important role in the pathophysiology of Alzheimer’s disease (AD) [1, 2]. Animal studies have shown that amyloid-β (Aβ) can disrupt synapses, and give rise to network hyperexcitability (NH), epileptiform discharges in local field potentials and EEG recordings, and epileptic seizures [3–5]. Hyperexcitability in its turn may promote further deposition of Aβ, creating a vicious circle [6]. The other pathological protein involved in AD, phosphorylated tau, has also been implied in disruption of the E/I balance [7]. These findings in animal models of AD have raised the question to what extent NH may also play a role in patients with AD. There is evidence that AD patients have a higher likelihood of suffering from epilepsy [8]. This prevalence may still be underestimated, since some types of seizure are difficult to detect clinically and may only be diagnosed with special electroencephalography (EEG) recordings [9]. NH may not only manifest itself as a higher likelihood of clinical seizures but may also be associated with a higher incidence of interictal epileptiform discharges (IEDs). There is now increasing evidence from EEG and magnetoencephalography (MEG) that IEDs occur more frequently in AD compared to healthy subjects [10]. AD patients with IEDs have a worse disease course with more rapid progression of cognitive decline that AD patients without IED [11]. An important question is whether IEDs in AD, even in the absence of clinical seizures, should be treated with anti-seizure medication. One small clinical trial with levetiracetam in AD could not show an effect on the primary endpoint but did reveal a positive effect on the Stroop interference naming subscale and the virtual route learning test in a subset of patients with IED [12].

There is an urgent need for sensitive and reliable biomarkers of hyperexcitability in AD, both for gaining a better understanding of the pathophysiology, but also to identify at an early-stage patients who might benefit from treatment directed at restoring the E/I balance. Epileptiform discharges in EEG or MEG recordings are the clinical gold standard for hyperexcitability, but their prevalence is low, and proper recognition is very much observer dependent. The yield may be increased with long term EEG monitoring, or the use of MEG instead of EEG, but these approaches are demanding for patients, expensive and not always generally available [11, 13–15]. Several alternative measures of NH which can be computed from EEG or MEG recordings have been proposed [16–18]. Functional connectivity (FC) are very promising candidates as biomarkers of NH since connections between distance brain regions consist mostly axons of excitatory neurons. In a recent MEG study, it was shown that AD patients with epileptiform discharges may have a higher FC in the low frequency bands (2–8 Hz) and a lower FC in the alpha band [19]. FC in the gamma band has also been related to epileptiform discharges in a MEG study in subjects with mild cognitive impairment (MCI) [20]. Studies using computational brain models have shown that systematic changes in the E/I balance are reflected in FC measures computed from simulated EEG/MEG time series [21, 22]. These studies suggest the potential value of FC as a biomarker of NH. However, the relation between E/I balance and FC is not straightforward, and may not be the same for all FC measures. Which FC measure is most sensitive to the earliest changes in the E/I balance in developing AD is currently unknown.

In the present study we want to compare the ability of four different FC measures to detect early phase AD-related neurophysiological abnormalities, in particular emerging hyperexcitability. As an objective benchmark for this comparison, we use the activity dependent degeneration (ADD) model of evolving structural and functional network changes in AD [23, 24]. This is a whole brain computational model which generates simulated EEG/MEG time series. AD-related structural and functional network changes are simulated by having high firing rates of excitatory neurons slowly weakening all synapses in the brain network. This ADD model can replicate many of the key features of developing abnormalities along the AD spectrum such as progressive slowing, a transient phase of hyperactivity and hyperconnectivity, and disruption of network architecture with preferential damage to highly connected hubs [23]. We aim to test the ability of FC biomarkers to distinguish between output of the ADD model and control data without ADD at different time points along the disease course. We compare this performance against relative theta power, which is sensitive to early changes in AD and has been related to a disturbed E/I balance in a recent model study [25].

We investigate the corrected amplitude envelope correlation (AECc) and the phase lag index (PLI) since these are well established FC measures with known test-retest reliability in EEG and MEG recordings of AD patients [26, 27]. In addition, we consider a recently introduced measure, the joint permutation entropy (JPE), which characterizes variability of local dynamics in addition to interregional connectivity [28]. A recent MEG study showed that the JPE performs well in comparison to the current gold standard of relative theta power in the classification of subjective cognitive decline versus MCI [29]. As a fourth biomarker we investigate a new measure, the phase lag time (PLT), a modification of the PLI which is less dependent upon epoch length and may be better able to capture rapid fluctuations in FC.

## MATERIALS AND METHODS

### The neural mass model

The brain is modeled as a network of coupled brain regions, or regions of interest (ROIs). The activity of each brain region is described with a neural mass model. We used a neural mass model originally introduced to describe the alpha rhythm and employed in many more recent studies to study a wide range of normal and pathological EEG and MEG phenomena [30–32]. Each neural mass consists of reciprocally coupled populations of excitatory and inhibitory neurons, which are characterized in terms of their mean membrane potentials and spike densities (spikes/s). In each population an impulse response function is used to convert the incoming spikes to changes in the mean membrane potential. This impulse response function is linear but has a memory which reflects the shape of excitatory and inhibitory post synaptic potentials. The mean membrane potential is subsequently converted to the outgoing spike density with a (static) nonlinear sigmoidal function. All neural masses receive input to their excitatory neurons from the thalamus. Time series of the mean membrane potential of the excitatory populations are used as the output of the model. These time series are assumed to reflect regionally generated EEG or MEG signals.

### Connections between neural masses

In agreement with previous studies, the excitatory neurons of the neural masses are connected to each other according to a structural connectivity matrix [31]. In the present study, in line we previous work, we used a network of 78 interconnected neural masses. The structural connectivity matrix was based upon the diffusion tractography data of a group of healthy subjects [33]. This is a binary graph where connections are either present or absent. In the model this unitary connection strength can be adjusted by multiplying it with a strength parameter S.

### Activity dependent degeneration algorithm

The ADD algorithm was introduced to simulate progressive changes in brain networks during the development of AD [23, 24]. The model rests upon a single assumption: excessive firing causes synaptic damage. This idea was implemented by computing for each neural mass at each point in time a loss function:

(1) $$\mathrm{loss}=e^{-d.\max\mathrm{Act}}$$

Here d is a parameter that determines the speed of degeneration and maxAct is the highest firing rate of the excitatory neurons in the last 20 time steps (of 2 ms.). Next, the strength of all synapses in the model (between excitatory and inhibitory populations in each neural mass; between thalamus and the excitatory neurons in all masses and between excitatory populations of all coupled neural masses) is decreased by multiplying it with loss.

### Running the model

**Fig. 1:**
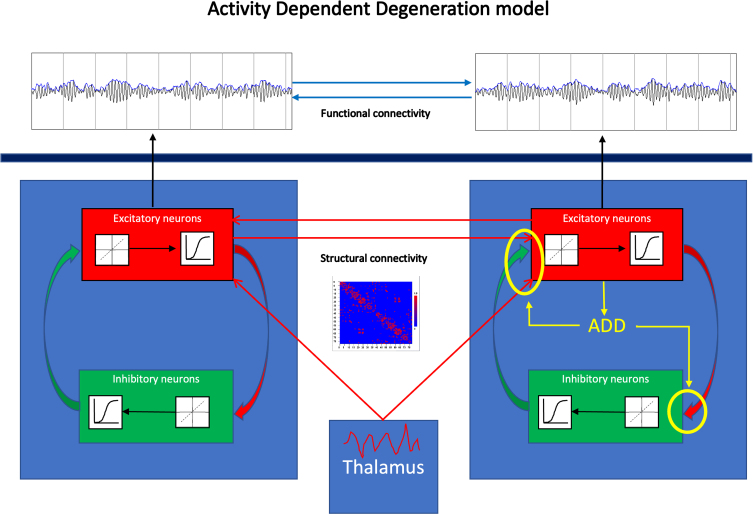
General scheme of the activity dependent degeneration (ADD) model. The brain is modeled as a network of coupled brain regions. The activity of each brain region, corresponding to the two blue squares to the left and the right, is described by a neural mass model. This considers the average activity (mean membrane potential and mean firing rates in spikes/s) of reciprocally interconnected populations of excitatory and inhibitory neurons, indicated by the red and green boxes within the larger blue boxes. Within each population incoming action potentials are converted to changes in mean membrane potential by a dynamic linear function. Membrane potentials are converted to spike densities by a static nonlinear (sigmoidal) function. Excitatory neurons of different brain regions are coupled to excitatory neurons of other brain regions, where the presence and strength of such connections is based upon an underlying structural connections matrix (example shown in the middle of the figure). All brain regions receive excitatory input to their excitatory neurons from the thalamus. The output of the model consists of time series of membrane potentials of the excitatory population of each of the brain regions. As shown in yellow in the right brain region ADD is implemented by coupling the firing rates of the excitatory population to weakening of all synapses, excitatory as well as inhibitory, present in the system.

To investigate the consequences of ADD the model is started with a set of initial parameter values which correspond to the healthy state. Next, a series of consecutive epochs is generated, corresponding to progressive disease induced changes. Each epoch has a length of 4,096 samples at a sample frequency of 500 Hz. For each epoch 5,000 samples are used for the system to reach a stable state. Note the presence of two separate time scales: within each epoch very small changes are happening every 2 ms according to formula (1). The numbers of subsequent epochs represent larger changes happening at longer timescale. It is known that the ADD model, implemented in this way, can explain several important features of progressive AD-related structural and functional network changes: (i) selective damage to highly connected “hub” nodes; (ii) progressive slowing characterized by decreasing frequency and increased power in low frequency bands; (iii) a transient state of increased firing rates of excitatory neurons, high oscillatory power and increased FC between the brain regions; (iv) a disruption of brain network organization [23]. In this study, the aim was to use the ADD as a benchmark to test and compare putative biomarkers for early-stage AD-related neurophysiological abnormalities. For this reason, we did not change anything to the original parameter settings of the model. A schematic overview of the model can be seen in Fig. 1. Mathematical details can be found in previous papers [23, 32]. An overview of the model parameters and the initial values is shown in Table 1.

**Table 1:** Overview of model parameters and initial values

Parameter	Interpretation	Initial value
Sample frequency		500 Hz
Sample time		2 ms
Epoch length		4096 samples
Pt	Thalamic input	550 spikes/s
Noiselevel	Fluctuations of thalamic input around mean	1.0
Amp1	Amplitude of EPSP	1.6 mV
Amp2	Amplitude of IPSP	32 mV
A1	Shape parameter of EPSP	55 s^-1^
B1	Shape parameter of EPSP	605 s^-1^
A2	Shape parameter of IPSP	27.5 s^-1^
B2	Shape parameter of IPSP	55 s^-1^
G	Shape parameter of sigmoidal function relating membrane potential to firing rates	25 s^-1^
Q	Shape parameter of sigmoidal function relating membrane potential to firing rates	0.34 mV^-1^
Vd1	Threshold potential for converting membrane potential to firing rates for excitatory neurons	7 mV
Vd2	Same as above for inhibitory neurons	7 mV
C1	Connection strength excitatory to inhibitory populations	32
C2	Connection strength inhibitory to excitatory populations	3
S	Coupling strength between neural masses	1.5
d	Speed of ADD process	0.01

EPSP, excitatory post synaptic potentials; IPSP, inhibitory post synaptic potentials; ADD, activity dependent degeneration.

### Functional connectivity measures

We investigated four FC measures for their performance as biomarkers of early-stage neurophysiological abnormalities in AD, in particular increasing NH, and compared these to relative theta power, since this has been shown to be one of the most reliable neurophysiological biomarkers in early AD [34, 35]. We selected the AECc and the PLI since these measures have been used in several previous studies and have been shown to be robust and reproducible in EEG as well as MEG recordings [26, 27]. To this we added the JPE which has been shown to perform better than the AECc and PLI, and at least as good as the relative theta power in a small previous study in subjects with subjective cognitive decline and MCI [29]. Finally, we also investigated a new measure, the PLT, a modification of the PLI which is better able to capture rapid fluctuations in connectivity and is less sensitive to epoch length.

As a first step data were filtered in the delta (0.5–4 Hz), theta (4–8 Hz), alpha (8–13 Hz), and beta (13–30 Hz) frequency bands. Next, for the computation of the AECc, PLI, and PLT, for each channel, the analytic signal was determined using a Hilbert transform as described previously [21, 36]. The analytical signal z_t_ is complex-valued with x_t_ a real time series and ${\tilde{x}}_{t}$ its corresponding Hilbert transform:

(2) $$z_{t}=x_{t}+i{\tilde{x}}_{t}=A_{t}e^{i\varphi_{t}}$$

The Hilbert transform of x_t_ is obtained via integration as follows:

(3) $${\tilde{x}}_{t}=\frac{1}{\pi }PV\int_{-x}^{\infty }\frac{x_{t}}{t-\tau }d\tau$$

where PV refers to the Cauchy principal value. The Hilbert transform [3] is related to the original signal by a [1/2]*π* phase shift that does not alter the spectral distribution (it can be computed by performing a Fourier transform, shifting all the phases by [1/2]*π*, followed by an inverse Fourier transform). From equation (2), both the instantaneous amplitude A_t_ and the instantaneous phase *φ*_t_ can be obtained:

(4) $$\varphi_{t}=\arctan\frac{y_{t}}{x_{t}}$$

where *φ*_t_ is the phase at time t (in radians) and

(5) $$\mathrm{amplitude}\;\mathrm{envelop}e_{t}=\sqrt{x_{t}^{2}+y_{t}^{2}}$$

is the amplitude envelope or instantaneous amplitude at time t. The (uncorrected) amplitude envelope correlation AEC is defined as the Pearson correlation of the amplitude envelopes of pairs of simulated time series. Correction for volume conduction can be done by pair-wise orthogonalization of the data before the AEC is computed [26, 27, 37]. We refer to this corrected version of the AEC as the AECc.

To compute the strength of phase synchronization between pairs of signals we used the phase lag index (PLI) which is not sensitive to volume conduction [36]:

(6) $$\mathrm{PL}I_{i,j}=\left|\mathrm{sign}\left[\sin\left(\varphi_{i,j}\right)\right]\right|$$

Here *sign* is the signum function which returns 1 if the argument if positive and –1 otherwise, and *φ*_i,j_ is the instantaneous phase difference between oscillators i and j. While the PLI is robust against detection of spurious of FC due to volume conduction, it is not optimal to capture rapidly fluctuating phase leading / lagging relations, and depends upon epoch length [38]. To deal with these problems we consider a modified measure, the phase lag time (PLT) which is defined as the average duration (in s) of phase leading / lagging relations between two signals. It is defined as follows:

(7) $$\mathrm{PL}T_{i,j}=1-e^{T}$$

Here T is the average duration (in sec.) of an interval between two successive sign changes of the phase difference. In the case of volume conduction, the phase difference and the PLT will be zero.

We also computed the joined permutation entropy (JPE) as a measure of FC that is also sensitive to variability of the local signals. For a detailed description of the measure, we refer to [28, 29]. Briefly, the computation of the JPE starts with representing the time series in each channel as a sequence of discrete ordinal patterns or symbols. Each pattern consists of n amplitude values separated by a time lag tau. As in our previous study we use a time lag of 1, and a pattern length of 4. Next, the n amplitudes in each pattern are ranked / ordered from the highest (1) to the lowest (n). This results in n! different possible ordinal patterns. In the case of two different signals, there are in total n! * n! different combinations of patterns. To correct for the spurious influence of volume conduction symmetric and anti-symmetric patterns in the two channels are excluded [39]. Finally, the JPE is computed as the Shannon information entropy of the resulting matrix of co-occurring ordinal patterns:

(8) H(n)=-∑p(π)log p(π)

Where n corresponds to the pattern length of 4, and the summation is over all entries of the matrix of co-occurring patterns, excluding the (anti) symmetric patterns. JPE was normalized between 0–1 by dividing it by its maximum value, i.e., log(n! * n! –2n!+1)^2^. As a last step, we inverted the sign to have a measure that increases with increased coupling strength and decreases with lower coupling strength:

(9) $$\mathrm{JP}E_{\mathrm{inv}}=-\frac{H\left(n\right)}{\log\left(n!*n!-2n!+1\right)}$$

For convenience in the rest of the paper we always assume the sign reversal, and use “JPE” to refer to the JPE_inv_.

### Software and statistical analysis

Modeling of the network of neural masses, implementation of the ADD algorithm, filtering of the simulated time series, computation of band power and all FC measures were done with BrainWave (version 1.2.12), which can be downloaded from https://github.com/CornelisStam/BrainWave.git. Statistical analyses (permutation tests; FDR; ROC plots, sensitivity, specificity, and accuracy) were also done with BrainWave.

## RESULTS

### Spectral features and evolution of E/I balance in the ADD model

In the present study, we aimed to use the ADD model as a benchmark for comparing a number of proposed FC biomarkers of early changes in AD in particular with respect to abnormalities in the E/I balance. The original paper introducing the model describes transient changes in total oscillatory power, firing rates of excitatory neurons and FC [23]. For the purpose of the present study more detailed information on changes in the underlying structural network, spectral properties, such as relative power in the theta band, and the actual E/I balance was needed to confirm its suitability as a benchmark.

**Fig. 2:**
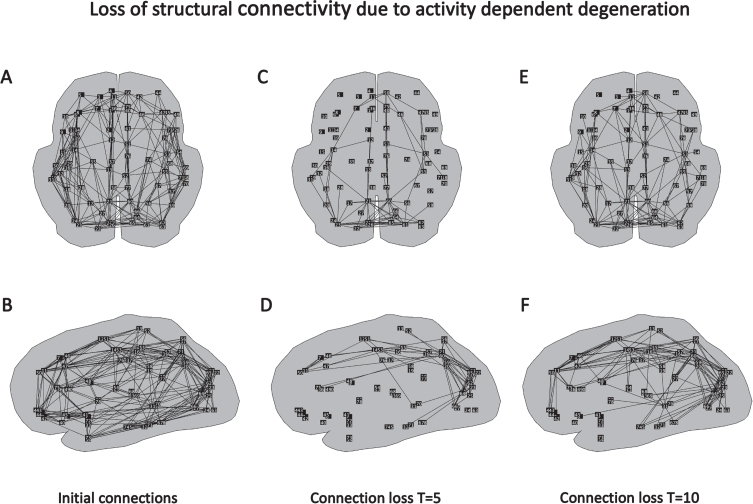
Spatial and temporal pattern of changes in structural connections in the global brain network induced by the ADD algorithm. A) Connections between brain regions in initial, healthy network, transversal view. B) Same network, sagittal view. C) Connections which have shown the strongest decrease in connection strength induced by the ADD algorithm at *T* = 5, transversal view. D) Same network, sagittal view. E) Connections with the strongest loss at *T* = 10, transversal view. F) Same network, sagittal view.

How the structural network changes due to the weakening of the connections guided by the ADD algorithm is shown in Fig. 2. In Fig. 2A and B, the initial, healthy structural network is shown in transversal and sagittal views. The strength of all initial connections is 1. Next, connections have been weakened under influence of the ADD algorithm (according to formula [1]). Figure 2C and D show which connections have been weakened most compared to the initial network. The largest damage can be seen in the posterior parietal and occipital areas, but the damage extends along the cingulum to the medial frontal regions. The situation at a later time, *T* = 10, is shown in Fig. 2E and F. Here we observe a further progress in weakening of connections in parietal occipital areas, cingulum, and medial frontal cortex. In addition, the damage now starts to extend into the temporal regions.

**Fig. 3:**
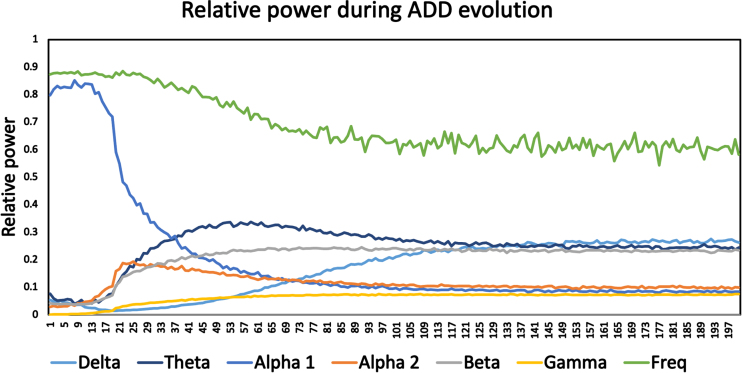
Time course of median frequency, and relative power in delta, theta, alpha1, alpha2, beta, and gamma bands during the activity dependent degeneration (ADD) disease/damage algorithm. Curves are based upon frequency analysis of the simulated 78-channel MEG time series of 200 epochs of a single run of the ADD model. The early, healthy state is characterized by a high median frequency, high power in the alpha1 band, and low power in the other bands. Over time the medians frequency decreases, the alpha1 power decreases, and power in the other frequency bands, in particular in the theta band, increases. After about 100 time steps a stable state is reached.

For this reason, the temporal evolution of relative power in a range of frequency bands as well as a measure of the dominant frequency were determined for the ADD model (Fig. 3). In the early phase, up to about epoch number 15, model output is dominated by oscillatory activity in the alpha 1 band (8–10 Hz) with a dominant frequency of about 8.5–9 Hz. In this phase relative power in the other bands is relatively low. Starting at about epoch 15 a number of changes occur: there is a slowing of the oscillatory activity with a decrease of the dominant frequency (to about 6–6.5 Hz), a strong decrease in alpha 1 power, and an increase in theta band power, from about 0.05 initially to more than 0.30 around epoch 50. Smaller increases are also observed in alpha 2, beta, gamma, and, at a later stage, delta power. The early increase in relative theta power, followed by a decrease in median frequency and a late increase in delta power are in agreement with spectral changes along the AD spectrum [40]. Changes in the higher frequency bands may be influenced by the emergence of a peak in the spectrum due to higher harmonics of the oscillatory activity.

**Fig. 4:**
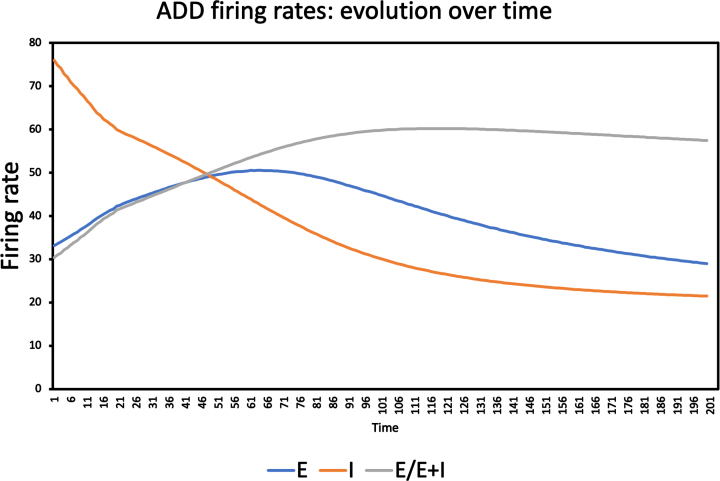
Time course of firing rates (spikes / sec.) of excitatory and inhibitory neurons, and E/I balance, averaged over all 78 neural masses, corresponding to the same model run as Fig. 3. The firing rate of the excitatory neurons shows an increase to a highest value around time step 60, followed by a gradual decline, and a final value below the initial value. The inhibitory neurons have much higher firing rates initially, and show a steady, approximately exponential decline before they reach a more or less table plateau at the end of the simulation. The E/I balance, computed as the ratio of the firing rates of excitatory neurons divided by the sum of excitatory and inhibitory firing rates, shows an almost linear increase from the start until it reaches a plateau after about 100 timesteps.

Next, we considered the temporal evolution of the firing rates of the excitatory neural populations (E) and the inhibitory neural populations (I). From these firing rates we computed a measure, normalized between 0 and 1, of the E/I balance as follows: E/(*E* + I). The results shown in Fig. 4 reproduced the initial increase (till about epoch 50–60) and subsequent decrease of excitatory firing rates, in agreement with the results reported in Fig. 5 of [23]. Somewhat surprisingly, we now observe that the inhibitory firing rates are initially much higher than the excitatory firing rates (75 spikes/s., as compared to 30 spikes/s for the excitatory population) and subsequently show a consistent, approximately exponential decrease. The measure of the E/I balance computed from these firing rates shows an almost linear increase from a value of 0.3 in the very beginning, until it reaches a plateau of about 0.6 at about epoch number 100, with only a minimal decrease afterwards (0.57 at epoch number 200). This is an important result since it shows that the activity-induced degeneration, as implemented in the original ADD model, does in fact induce an early, and progressive increase in the E/I balance. This, in combination with the increase in theta and delta power, and the decrease in dominant frequency, makes the ADD model a useful benchmark to investigate the performance of proposed biomarkers for early AD-related neurophysiological abnormalities. Further support for the validity of the ADD model based upon a comparison to empirical MEG recordings indifferent stages along the AD continuum can be found in the Supplementary Material.

**Fig. 5:**
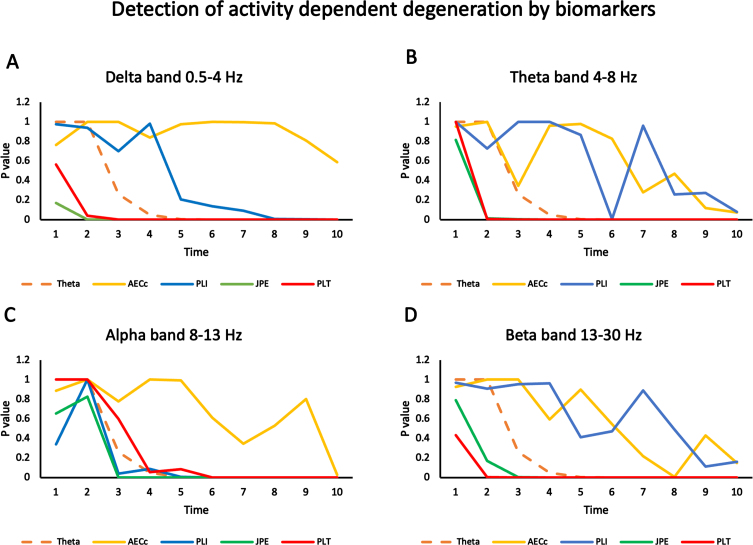
Sensitivity of biomarkers to detect progressive changes over time in ADD model. Statistical differences in several biomarkers (relative theta power, AECc, PLI, JPE, and PLT) are computed for two data sets (ADD: 20 files corresponding to separate runs of the ADD algorithm and 20 files of normal activity without ADD), for time steps from 1 to 10. The significance (p-value of difference in mean over all ROIs, FDR corrected) is plotted as a function of time step for all measures and 10 timesteps in four frequency bands. A) Results for the delta band (0.5-4 Hz). Results for relative theta power are shown in all plots with a striped line for reference. AECc shows no significant results for any time step. PLI is significant from timestep 8 on. JPE and PLT are significant from timestep 2 on. B) Results for the theta band (4-8 Hz). The curves of AECc and PLI show a declining trend for later timesteps but no significant effects with the exception of the PLI for timestep 6. Both JPE and PLT are significant from timestep 2 on. C) Results for the alpha band (8-13 Hz). The AECc only shows a significant effect at timestep 10. The PLI first becomes significant from timestep 6, the JPE from timestep 3 and the PLT from timestep 6. D) Results for the beta band (13-30 Hz). The AECc and the PLI show a declining trend but no significant results except for the AECc at timestep 8. The JPE is significant from timestep 3 and the PLT from timestep 2 on.

### Sensitivity of FC biomarkers to changes in the ADD model

Twenty independent runs of the ADD model (ADD condition) and 20 independent runs without activity induced synaptic changes (Control condition ‘Con’) were used to compare the performance of the four FC measures: AECc, PLI, JPE, and PLT. For each measure we computed the significance of the difference between the ADD and Con files as a function of time (number of epochs since start of the model, where higher epoch number corresponds to a more progressed / later disease stage). For comparison we also include the performance of relative theta power which has been shown to be one of the most robust biomarkers of early-stage AD [34]. The main results are shown in Fig. 5. In the delta band the AECc did not detect any significant differences between the ADD and Con conditions. The PLI could detect the difference from epoch 8 onwards. Relative theta power could detect the changes in the ADD condition from epoch 5 onwards. JPE already detected the difference from epoch 2 onwards, and the PLT from epoch 3 onwards. A very similar pattern was observed for the theta band (Fig. 5B). Here the AECc and PLI did not detect the difference between ADD and Con conditions, with one outlier for the PLI in epoch 6. In contrast, both JPE and PLT detected the difference between ADD and Con from epoch 2 onwards. For the alpha band the results were slightly different: the AECc only detected the difference at epoch 10. The PLT was slightly worse than the relative theta power and detected the difference from epoch 6 on. PLI and JPE behaved very similar and showed significant results from epoch 3 on. Finally, the beta band showed a pattern rather similar to the delta and theta bands: AECc (with an outlier in epoch 8) and PLI were not able to detect differences between the ADD and Con conditions, while the JPE showed a significant difference from epoch 3 on, and the PLT from epoch 2 on. Overall, the results show that the classic measures AECc and PLI detect the ADD process at a later stage than the newer measures JPE and PLT in most frequency bands. Performance of relative theta power is intermediated between the classic measures on the one hand, and the new measures on the other hand.

**Fig. 6:**
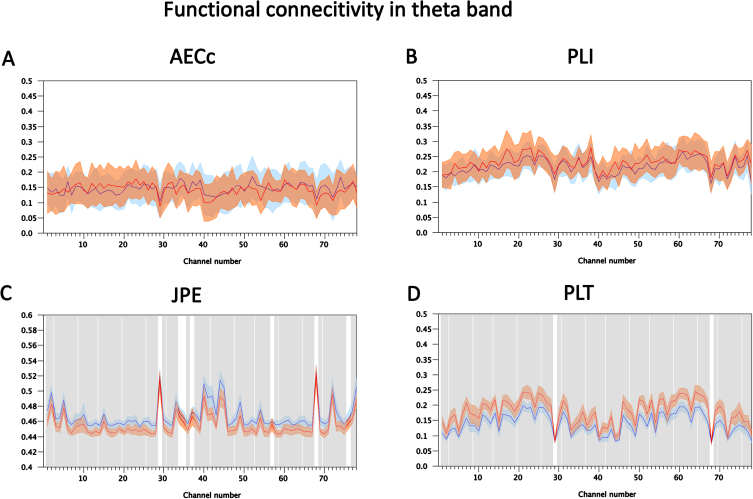
Detailed results for FC markers in theta band (4-8 Hz) at timestep *T* = 5. Shown are the mean values (shading 2 standard errors of the mean) of functional connectivity measures for each of the 78 ROIs for the set of 20 ADD and 20 Con files. A) Results for AECc. No significant differences between ADD and Con after FDR correction. B) Results for the PLI. No significant differences between ADD and Con after FDR correction. C) Results for the JPE. The blue curve, corresponding to the ADD group, shows higher values than the red curve, corresponding to the Con group. Significant differences after FDR correction are indicated with gray shading. D) Results for the PLT. The ADD group has lower values than the Con group (red line). Significant differences after FDR correction are indicated with gray shading.

**Fig. 7:**
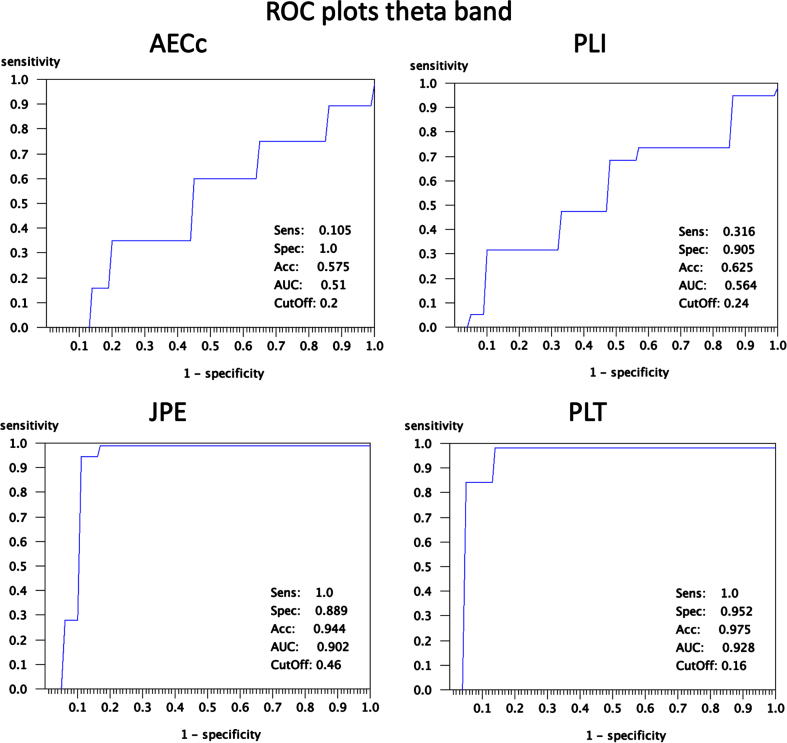
ROC plots corresponding to Fig. 5. For each of the four functional connectivity measures the sensitivity of the mean connectivity is plotted as a function of 1-specificity. Sensitivity, specificity and accuracy at the point of maximal accuracy are shown in each plot in the lower right corner. The area under the curve and the cutoff value at the point of maximum accuracy are also shown in the lower right corner.

To illustrate the spatial distribution, direction and magnitude of these effects more detailed results for the theta band at epoch 5, where the results for the different measures are clearly diverging, are shown in Figs. 6 and 7. Figure 6 shows for all four measures and for all 78 AAL ROIs examined the mean value (2 * SEM in shading) of the FC measures (blue = ADD, red = Con). Gray shading corresponds to significant differences (permutation tests; FDR corrected for number of ROIs) at the ROI level. For the AECc and PLI there is an almost complete overlap between the ADD and Con conditions, with no significant differences. For the JPE we see a significantly higher FC for the ADD group in almost all ROIs, with a few exceptions (notably AAL region 29/68: Heschl’s gyrus). For the PLT the ADD condition shows lower FC for almost all regions, again with a few exceptions (notably AAL region 29/68: Heschl’s gyrus). The ROC plots corresponding to Fig. 6 are shown in Fig. 7. At the optimal cut-off the AECc has a sensitivity of 0.105, a specificity of 1.0, and accuracy of 0.575 and an area under the curve (AUC) of 0.51. The PLI has a sensitivity of 0.316, a specificity of 0.905, an accuracy of 0.625 and an AUC of 0.564 at the optimal cut-off of 0.24. The JPE has a sensitivity of 1.0, a specificity of 0.889, and accuracy of 0.944 and an AUC of 0.902 at the optimal cut-off of 0.46. Finally, at an optimal cut-off of 0.16 the PLT has a sensitivity of 1.0, a specificity of 0.952, an accuracy of 0.975 and an AUC of 0.928. The analysis with ROC plots thus confirms that the AECc and PLI perform hardly above chance level in detecting early neurophysiological abnormalities in the ADD condition, whereas the JPE and PLT show very good performance in classifying the ADD and Con conditions.

## DISCUSSION

The aim of the present study was to compare a number of FC measures which could be used as biomarkers of disturbed E/I balance in early AD. We showed that a previously introduced ADD model of early AD displays the progressive slowing of the oscillatory activity and the progressive increase of the E/I balance, and thus could serve as a benchmark for testing the FC biomarkers. Compared to relative theta power, frequently used FC measures such as the AECc and PLI performed worse in detecting the progressive abnormalities in the ADD model compared to a healthy reference state. In contrast, the JPE and the PLT, a proposed improvement of the PLI, generally performed better in detecting early neurophysiological abnormalities compared to the AEC and PLI, as well as the relative theta power. These results suggest first that novel measures, such as the JPE and PLT are promising biomarkers for detecting early-stage neurophysiological abnormalities, in particular NH. In addition, we have demonstrated that using a computational model of progressive structural and functional network changes in AD could be an objective and effective way to compare future new biomarkers.

### Usefulness of the ADD model as a benchmark for comparing FC measures

In the present study we were interested in comparing the performance of FC biomarkers in detecting neurophysiological abnormalities, and in particular NH in the earliest possible disease stages using a whole brain computational model. The ADD model assumes that excessive firing of excitatory neurons progressively damages all synapses in neural networks [23]. As has been shown previously, this simple scenario can reproduce many of the key features of progressive structural and functional network changes in AD such as progressive loss of structural connections, slowing of oscillatory activity, a transient phase of increased activity and FC, and disruption of spatial temporal patterns of network organization with preferential damage of highly connected hub nodes [23]. However, in the original study a transient increase in firing was reported only for excitatory neurons, and changes in different frequency bands over time were not described in detail. In the present study we show that the ADD model produces a gradual decrease in median frequency and relative alpha1 power, and an increase in theta and delta power which are in line with spectral changes along the AD spectrum [41]. Furthermore, by taking both excitatory as well as inhibitory firing rates into account, we could now show that the E/I balance in the model shows a steady increase from the beginning, until a steady state is reached in a fairly late stage. This confirms that ADD is a good model of progressive NH. It should be noted that the increasing E/I balance is an emergent property of the ADD scenario, and not simply the result of setting an explicit E/I balance parameter. Other whole brain models of AD have been proposed in recent years [21, 42–45]. In these models, parameters were often chosen based upon a fit of the model to empirical functional and structural connectivity, and sometimes data on of pathological proteins in a small number of states along the AD spectrum. While these models provide insight in possible mechanisms, often they do not have the high temporal resolution of the ADD model and are therefore less suitable as a testbench for the performance of FC biomarkers of hyperexcitability.

### Amplitude envelope correlation

Compared to other measures of FC, the AEC is often considered to be one of the most robust measures of FC in EEG and MEG data, in particular if it is corrected for volume conduction [37, 46, 47]. The AECc shows a clear and reproducible loss of FC in the alpha and beta, but not the theta band in AD patients compared to subjects with subjective cognitive decline [26, 27]. It has been suggested that the AECc could be a biomarker that shows abnormalities in the earliest stages of AD [48]. However, in the present study the AECc did not perform very well compared to relative theta power and other FC measures. In the delta band the AECc never detected the differences between the ADD and Con data, and in the other bands it only detected differences at a much later time point than the other measures. It is not yet clear what caused the disappointing performance of the AECc in the present study. We used the AECc with correction for volume conduction, even though the ADD model generates time series for each region without any volume conduction effects. Use of the uncorrected AEC might have improved the performance but would make it more difficult to relate the results to those of empirical studies which mostly use the AECc. It could be that the relatively short epochs (8.192 sec.) in the present study were less optimal in combination with the AEC [27, 38]. Other features of the ADD model, such as the choice of the structural connectivity matrix, and absence of conduction delay between the brain regions, and the choice of the neural mass model and its initial parameter settings could also play a role. However, all measures were tested on the exact same model, so it remains unclear why the AECc would perform less than the other measures. While this problem should be addressed in future studies, for now the conclusion is that the AECc is less sensitive to the earliest changes in AD than relative theta power and other FC biomarkers.

### Phase lag index

The PLI is a measure of phase synchronization that is corrected for volume conduction or field spread. Several studies have shown changes in FC in AD and MCI, in particular in the alpha and beta band, using the PLI [49, 50]. Compared to the AEC the PLI may be better in reproducibly detecting increased connectivity in the theta band [26, 27]. However, compared to other FC measures the PLI may be more sensitive to noise and the effects of epoch length [38, 46, 51]. Of note, the weighted PLI, a corrected version of the PLI that was introduced to make the measure less sensitive to noise, suffers from the problem that it reflects both the strength of the phase synchronization as well as the magnitude of the phase difference [52]. In the present study the PLI performed slightly better than the AECc, but worse than the relative theta power and the other FC measures in all bands except the alpha band. In the alpha band the PLI performed comparable to all other measures with the exception of the AEC which performed worse than all other measures. The relatively short epochs in the present study could explain perhaps part of the superior performance of the PLI compared to the AECc. In view of the sensitivity to noise of the PLI it can be understood why its performance is optimal in the alpha band, where the signal to noise ratio is highest in the early stages of the ADD scenario. The inferior performance of the PLI compared to the JPE and PLT could be due to its inability to capture rapid fluctuations in FC, which will be discussed in more detail below.

### Joint permutation entropy

The JPE is a recently introduced measure which is sensitive to both the strength of the coupling between two time series (corrected for volume conduction) as well as the variability of the individual signals [28, 29]. The motivation for this measure is the idea that signal variability might reflect the E/I balance of the underlying neural networks [53]. In a pilot study the JPE performed at least as good as the current gold standard, relative theta power, in the classification of resting-state MEG recordings in the theta band of subjects with subjective cognitive decline or subjects with MCI [29]. The present study lends further support for the possible usefulness of the JPE as a biomarker of early changes in AD. The JPE could detect differences between the ADD and the Con condition at an earlier stage than all other measures in all frequency bands (with the exception of the PLT in the beta band). In the theta band at timestep 5 the JPE has a sensitivity of 1.0, a specificity of 0.889, and an accuracy of 0.994. These test characteristics are very promising for a biomarker, but they are likely to be overly optimistic since they were obtained in a computer simulation under perfectly controlled conditions. A disadvantage of the JPE and related measures based upon ordinal patterns is that they require the choice of several parameters, in particular the time-delay *tau* and embedding dimension *d*. In the present study we have restricted ourselves to the parameter choice used in our previous study [29]. However, it is not known whether this choice is optimal, and whether different choices have to be made for different frequency bands.

### Phase lag time

The PLI is a reliable measure of phase synchronization corrected for volume conduction, but it is sensitive to noise and may be less suitable to capture rapid fluctuations in phase leading/lagging relations between time series. Here we considered a modification of the PLI, the PLT, which is the time between two consecutive zero crossing/sign changes of the instantaneous phase difference. This parameter-free measure is related to other measures that capture rapid changes in phase synchronization [54–56]. An in-depth description of the measure will be given in a forthcoming paper. Here we focus on the potential of the PLT as a biomarker for early changes in AD. In almost all frequency bands with the exception of the alpha band the PLT was better than the AECc, PLI and relative theta power, and slightly worse or slightly better (in the beta band) compared to the JPE. In the alpha band the PLT was much better than the AEC, and only slightly worse than the other measures. In the theta band for time step 5 the PLT had very good test characteristics: sensitivity = 1.0, specificity = 0.952, and accuracy = 0.975. This classification performance is at the same level as that of the JPE, and much better than that of the AECc and PLI. Importantly, the PLT always seems to perform substantially better than the PLI from which it is derived. This suggests that this modification which helps to deal with rapid fluctuations in phase leading/lagging relations improves performance as a biomarker for early AD and NH. However, while these results are promising, they need to be confirmed by application of the measure to empirical recordings of subjects in the earliest phases of the AD spectrum.

### Strengths and weaknesses

An advantage of using a computational model of progressive neurophysiological abnormalities for testing and comparing candidate biomarkers is the fact that we have exact control over the gold standard. All the relevant properties of the model, such as the exact E/I balance, are known at all time points. Such a level of control over the gold standard is difficult to achieve in empirical datasets of subjects along the AD spectrum, where for instance the true E/I balance is not known. The downside of using a model as a benchmark is that all models are unavoidably gross simplifications of the nature and variability of the actual pathological processes underlying progressive AD. Another limitation, in addition to the level of biological detail is that the specific type of model used in this study, a network of coupled neural masses, could have influenced the results. A challenge for future work is to improve the model by investigating which level of detail is necessary, and which mechanisms—such as ADD—have to be assumed in order to replicate empirically observed properties of structural and functional brain networks along the AD spectrum. Obvious targets for future improvements are the resolution of the brain atlas, the choice of the structural connectome and the inclusion of volume conduction and time delays. Another interesting extension would be the incorporation of some mechanism of plasticity, as previously proposed to explain the emergence of modular structure of brain networks and the recovery after brain damage [57]. However, whether in its present form, or after future improvements, we have shown that the ADD model presents a useful benchmark for testing putative biomarkers for early AD-related neurophysiological abnormalities and disturbed E/I balance. The present study shows that the JPE and PLT are promising biomarkers of early neurophysiological changes in AD, and in particular NH, which should be further explored in clinical datasets

### Conclusions

In conclusion we have shown how a whole-brain computational model of progressive structural and functional network changes in AD can be used as an objective benchmark for the comparison of proposed biomarkers for NH in the earliest disease stage. We could replicate the observation from empirical studies that AECc and PLI are less sensitive to early neurophysiological abnormalities than relative theta power. However, we could also show that novel measures such as the JPE and the PLT, introduced in this study, display superior performance in detecting early-stage neurophysiological abnormalities. These novel FC biomarkers are promising candidates for use in empirical studies aimed at detecting the earliest phase of NH.

## AUTHOR CONTRIBUTIONS

Cornelis Stam (Conceptualization; Data curation; Formal analysis; Investigation; Methodology; Software; Visualization; Writing – original draft; Writing – review & editing); Willem de Haan (Conceptualization; Data curation; Formal analysis; Investigation; Resources; Software; Writing – original draft; Writing – review & editing).

## Supplementary Material

Supplementary Material

## Data Availability

The datasets analyzed in the study were all generated with the BrainWave software, written by the first author, and freely available from: https://github.com/CornelisStam/BrainWave.git.

## References

[1] Babiloni C (2022) The dark side of Alzheimer’s disease: Neglected physiological biomarkers of brain hyperexcitability and abnormal consciousness level. J Alzheimers Dis 88, 801–807. 35754282 10.3233/JAD-220582

[2] Maestú F , de Haan W , Busche MA , DeFelipe J (2021) Neuronal excitation/inhibition imbalance: Core element of a translational perspective on Alzheimer pathophysiology. Ageing Res Rev 69, 101372. 34029743 10.1016/j.arr.2021.101372

[3] Busche MA , Konnerth A (2016) Impairments of neural circuit function in Alzheimer’s disease. Philos Trans R Soc Lond B Biol Sci 371, 20150429. 27377723 10.1098/rstb.2015.0429 PMC4938029

[4] Cirrito JR , Yamada KA , Finn MB , Sloviter RS , Bales KR , May PC , Schoepp DD , Paul SM , Mennerick S , Holtzman DM (2005) Synaptic activity regulates interstitial fluid amyloid-beta levels *in vivo*.. Neuron 48, 913–22. 16364896 10.1016/j.neuron.2005.10.028

[5] Tok S , Ahnaou A , Drinkenburg W (2022) Functional neurophysiological biomarkers of early-stage Alzheimer’s disease: A perspective of network hyperexcitability in disease progression. J Alzheimers Dis 88, 809–836. 34420957 10.3233/JAD-210397 PMC9484128

[6] Tombini M , Assenza G , Ricci L , Lanzone J , Boscarino M , Vico C , Magliozzi A , Di Lazzaro V (2021) Temporal lobe epilepsy and Alzheimer’s disease: From preclinical to clinical evidence of a strong association. J Alzheimers Dis Rep 5, 243–261. 34113782 10.3233/ADR-200286 PMC8150253

[7] Wu JW , Hussaini SA , Bastille IM , Rodriguez GA , Mrejeru A , Rilett K , Sanders DW , Cook C , Fu H , Boonen RA , Herman M , Nahmani E , Emrani S , Figueroa YH , Diamond MI , Clelland CL , Wray S , Duff KE (2016) Neuronal activity enhances tau propagation and tau pathology*in vivo*. Nat Neurosci 19, 1085–1092. 27322420 10.1038/nn.4328 PMC4961585

[8] Horváth A , Szűcs A , Barcs G , Noebels JL , Kamondi A (2016) Epileptic seizures in Alzheimer disease: A review. AlzheimerDis Assoc Disord 30, 186–192. 10.1097/WAD.0000000000000134 26756385

[9] Lam AD , Deck G , Goldman A , Eskandar EN , Noebels J , Cole AJ (2017) Silent hippocampal seizures and spikes identified by foramen ovale electrodes in Alzheimer’s disease. Nat Med 23, 678–680. 28459436 10.1038/nm.4330 PMC5461182

[10] Csernus EA , Werber T , Kamondi A , Horvath AA (2022) The significance of subclinical epileptiform activity in Alzheimer’s disease: A review. Front Neurol 13, 856500. 35444602 10.3389/fneur.2022.856500 PMC9013745

[11] Vossel KA , Ranasinghe KG , Beagle AJ , Mizuiri D , Honma SM , Dowling AF , Darwish SM , Van Berlo V , Barnes DE , Mantle M , Karydas AM , Coppola G , Roberson ED , Miller BL , Garcia PA , Kirsch HE , Mucke L , Nagarajan SS (2016) Incidence and impact of subclinical epileptiform activity in Alzheimer’s disease. Ann Neurol 80, 858–870. 27696483 10.1002/ana.24794 PMC5177487

[12] Vossel K , Ranasinghe KG , Beagle AJ , La A , Ah Pook K , Castro M , Mizuiri D , Honma SM , Venkateswaran N , Koestler M , Zhang W , Mucke L , Howell MJ , Possin KL , Kramer JH , Boxer AL , Miller BL , Nagarajan SS , Kirsch HE (2021) Effect of levetiracetam on cognition in patients with Alzheimer disease with and without epileptiform activity: A randomized clinical trial. JAMA Neurol 78, 1345–1354. 34570177 10.1001/jamaneurol.2021.3310 PMC8477304

[13] Horvath AA , Papp A , Zsuffa J , Szucs A , Luckl J , Radai F , Nagy F , Hidasi Z , Csukly G , Barcs G , Kamondi A (2021) Subclinical epileptiform activity accelerates the progression of Alzheimer’s disease: A long-term EEG study. Clin Neurophysiol 132, 1982–1989. 34034963 10.1016/j.clinph.2021.03.050

[14] Lam AD , Shafi MM (2022) Towards a coherent view of network hyperexcitability in Alzheimer’s disease. Brain 145, 423–425. 35259227 10.1093/brain/awac033

[15] Musaeus CS , Frederiksen KS , Andersen BB , Høgh P , Kidmose P , Fabricius M , Hribljan MC , Hemmsen MC , Rank ML , Waldemar G , Kjær TW (2023) Detection of subclinical epileptiform discharges inAlzheimer’s disease using long-term outpatient EEG monitoring. Neurobiol Dis 183, 106149. 37196736 10.1016/j.nbd.2023.106149

[16] Ahmad J , Ellis C , Leech R , Voytek B , Garces P , Jones E , Buitelaar J , Loth E , Dos Santos FP , Amil AF , Verschure PFMJ , Murphy D , McAlonan G (2022) From mechanisms to markers: Novel noninvasive EEG proxy markers of the neural excitation and inhibition system in humans. Transl Psychiatry 12, 467. 36344497 10.1038/s41398-022-02218-z PMC9640647

[17] Cope ZA , Murai T , Sukoff Rizzo SJ (2022) Emerging electroencephalographic biomarkers to improve preclinical to clinical translation in Alzheimer’s disease. Front Aging Neurosci 14, 805063. 35250541 10.3389/fnagi.2022.805063 PMC8891809

[18] Joseph S , Patterson R , Wang W , Blumberger DM , Rajji TK , Kumar S ( (2022) Quantitative assessment of cortical excitability in Alzheimer’s dementia and its association with clinical symptoms: A systematic review and meta-analyses. J Alzheimers Dis 88, 867–891. 34219724 10.3233/JAD-210311

[19] Ranasinghe KG , Kudo K , Hinkley L , Beagle A , Lerner H , Mizuiri D , Findlay A , Miller BL , Kramer JH , Gorno-Tempini ML , Rabinovici GD , Rankin KP , Garcia PA , Kirsch HE , Vossel K , Nagarajan SS (2022) Neuronal synchrony abnormalities associated with subclinical epileptiform activity in early-onset Alzheimer’s disease. Brain 145, 744–753. 34919638 10.1093/brain/awab442 PMC9630715

[20] Cuesta P , Ochoa-Urrea M , Funke M , Hasan O , Zhu P , Marcos A , López ME , Schulz PE , Lhatoo S , Pantazis D , Mosher JC , Maestu F (2022) Gamma band functional connectivity reduction in patients withamnestic mild cognitive impairment and epileptiform activity. Brain Commun 4, fcac012. 35282163 10.1093/braincomms/fcac012 PMC8914494

[21] Stam CJ , van Nifterick AM , de Haan W , Gouw AA (2023) Network hyperexcitability in early Alzheimer’s disease: Is functional connectivity a potential biomarker? Brain Topogr 36, 595–612. 37173584 10.1007/s10548-023-00968-7 PMC10293463

[22] van Nifterick AM , Scheijbeler EP , Gouw AA , de Haan W , Stam CJ (2024) Local signal variability and functional connectivity: Sensitive measures of the excitation-inhibition ratio? Cogn Neurodyn 18, 519–537. 38699618 10.1007/s11571-023-10003-x PMC11061092

[23] de Haan W , Mott K , van Straaten EC , Scheltens P , Stam CJ (2012) Activity dependent degeneration explains hub vulnerability in Alzheimer’s disease. PLoS Comput Biol 8, e1002582. 22915996 10.1371/journal.pcbi.1002582 PMC3420961

[24] de Haan W , van Straaten ECW , Gouw AA , Stam CJ (2017) Altering neuronal excitability to preserve network connectivity in a computational model of Alzheimer’s disease. PLoS Comput Biol 13, e1005707. 28938009 10.1371/journal.pcbi.1005707 PMC5627940

[25] van Nifterick AM , Gouw AA , van Kesteren RE , Scheltens P , Stam CJ , de Haan W (2022) A multiscale brain network model links Alzheimer’s disease-mediated neuronal hyperactivity to large-scale oscillatory slowing. Alzheimers Res Ther 14, 101. 35879779 10.1186/s13195-022-01041-4 PMC9310500

[26] Briels CT , Schoonhoven DN , Stam CJ , de Waal H , Scheltens P , Gouw AA (2020) Reproducibility of EEG functional connectivity in Alzheimer’s disease. Alzheimers Res Ther 12, 68. 32493476 10.1186/s13195-020-00632-3 PMC7271479

[27] Schoonhoven DN , Briels CT , Hillebrand A , Scheltens P , Stam CJ , Gouw AA (2022) Sensitive and reproducible MEG resting-state metrics of functional connectivity in Alzheimer’s disease. Alzheimers Res Ther 14, 38. 35219327 10.1186/s13195-022-00970-4 PMC8881826

[28] Yin Y , Shang P , Ahn AC , Peng CK (2019) Multiscale joint permutation entropy for complex time series. Physica A 515, 388–402.

[29] Scheijbeler EP , van Nifterick AM , Stam CJ , Hillebrand A , Gouw AA , de Haan W (2022) Network-level permutation entropy of resting-state MEG recordings: A novel biomarker for early-stage Alzheimer’s disease? Netw Neurosci 6, 382–400. 35733433 10.1162/netn_a_00224 PMC9208018

[30] Lopes da Silva FH , Hoeks A , Smits H , Zetterberg LH (1974) Model of brain rhythmic activity. The alpha-rhythm of the thalamus. Kybernetik 15, 27–37. 4853232 10.1007/BF00270757

[31] Ponten SC , Tewarie P , Slooter AJ , Stam CJ , van Dellen E (2013) Neural network modeling of EEG patterns in encephalopathy. J Clin Neurophysiol 30, 545–52. 24084188 10.1097/WNP.0b013e3182a73e16

[32] Stam CJ , Pijn JP , Suffczynski P , Lopes da Silva FH (1999) Dynamics of the human alpha rhythm: Evidence for non-linearity? Clin Neurophysiol 110, 1801–1813. 10574295 10.1016/s1388-2457(99)00099-1

[33] Gong G , He Y , Concha L , Lebel C , Gross DW , Evans AC , Beaulieu C (2009) Mapping anatomical connectivity patterns of human cerebral cortex using *in vivo* diffusion tensor imaging tractography. Cereb Cortex 19, 524–536. 18567609 10.1093/cercor/bhn102 PMC2722790

[34] Gouw AA , Alsema AM , Tijms BM , Borta A , Scheltens P , Stam CJ , van der Flier WM (2017) EEG spectral analysis as a putative early prognostic biomarker in nondemented, amyloid positive subjects. Neurobiol Aging 57, 133–142. 28646686 10.1016/j.neurobiolaging.2017.05.017

[35] Gouw AA , Hillebrand A , Schoonhoven DN , Demuru M , Ris Scheltens P , Stam CJ (2021) Routine magnetoencephalography in memory clinic patients: A machine learning approach. Alzheimers Dement 13, e12227. 10.1002/dad2.12227 PMC8449227 34568539

[36] Stam CJ , Nolte G , Daffertshofer A (2007) Phase lag index: Assessment of functional connectivity from multi channel EEG and MEG with diminished bias from common sources. Hum Brain Mapp 28, 1178–1193. 17266107 10.1002/hbm.20346 PMC6871367

[37] Hipp JF , Hawellek DJ , Corbetta M , Siegel M , Engel AK (2012) Large-scale cortical correlation structure of spontaneous oscillatory activity. Nat Neurosci 15, 884–890. 22561454 10.1038/nn.3101 PMC3861400

[38] Fraschini M , Demuru M , Crobe A , Marrosu F , Stam CJ , Hillebrand A (2016) The effect of epoch length on estimated EEG functional connectivity and brain network organisation. J Neural Eng 13, 036015. 27137952 10.1088/1741-2560/13/3/036015

[39] King JR , Sitt JD , Faugeras F , Rohaut B , El Karoui I , Cohen L , Naccache L , Dehaene S (2013) Information sharing in the brain indexes consciousness in noncommunicative patients. Curr Biol 23, 1914–1919. 24076243 10.1016/j.cub.2013.07.075 PMC5635964

[40] Babiloni C , Arakaki X , Azami H , Bennys K , Blinowska K , Bonanni L , Bujan A , Carrillo MC , Cichocki A , de Frutos-Lucas J , Del Percio C , Dubois B , Edelmayer R , Egan G , Epelbaum S , Escudero J , Evans A , Farina F , Fargo K , Fernández A , Ferri R , Frisoni G , Hampel H , Harrington MG , Jelic V , Jeong J , Jiang Y , Kaminski M , Kavcic V , Kilborn K , Kumar S , Lam A , Lim L , Lizio R , Lopez D , Lopez S , Lucey B , Maestú F , McGeown WJ , McKeith I , Moretti DV , Nobili F , Noce G , Olichney J , Onofrj M , Osorio R , Parra-Rodriguez M , Rajji T , Ritter P , Soricelli A , Stocchi F , Tarnanas I , Taylor JP , Teipel S , Tucci F , Valdes-Sosa M , Valdes-Sosa P , Weiergräber M , Yener G , Guntekin B (2021) Measures of resting state EEG rhythms for clinicaltrials in Alzheimer’s disease: Recommendations of an expert panel. Alzheimers Dement 17, 1528–1553. 33860614 10.1002/alz.12311 PMC8647863

[41] Babiloni C , Blinowska K , Bonanni L , Cichocki A , De Haan W , DelPercio C , Dubois B , Escudero J , Fernández A , Frisoni G , Guntekin B , Hajos M , Hampel H , Ifeachor E , Kilborn K , Kumar S , Johnsen K , Johannsson M , Jeong J , LeBeau F , Lizio R , Lopes da Silva F , Maestú F , McGeown WJ , McKeith I , Moretti DV , Nobili F , Olichney J , Onofrj M , Palop JJ , Rowan M , Stocchi F , Struzik ZM , Tanila H , Teipel S , Taylor JP , Weiergräber M , Yener G , Young-Pearse T , Drinkenburg WH , Randall F (2020) What electrophysiology tells usabout Alzheimer’s disease: A window into the synchronization andconnectivity of brain neurons. Neurobiol Aging 85, 58–73. 31739167 10.1016/j.neurobiolaging.2019.09.008

[42] Alexandersen CG , de Haan W , Bick C , Goriely A (2023) A multi-scale model explains oscillatory slowing and neuronal hyperactivity in Alzheimer’s disease. J R Soc Interface 20, 20220607. 36596460 10.1098/rsif.2022.0607 PMC9810432

[43] Demirtaǯ M , Falcon C , Tucholka A , Gispert JD , Molinuevo JL , Deco G (2017) A whole-brain computational modeling approach to explainthe alterations in resting-state functional connectivity duringprogression of Alzheimer’s disease. Neuroimage Clin 16, 343–354. 28861336 10.1016/j.nicl.2017.08.006 PMC5568172

[44] Ranasinghe KG , Verma P , Cai C , Xie X , Kudo K , Gao X , Lerner H , Mizuiri D , Strom A , Iaccarino L , La Joie R , Miller BL , Gorno-Tempini ML , Rankin KP , Jagust WJ , Vossel K , Rabinovici GD , Raj A , Nagarajan SS (2022) Altered excitatory and inhibitory neuronal subpopulation parameters are distinctly associated with tau and amyloid in Alzheimer’s disease. Elife 11, e77850. 35616532 10.7554/eLife.77850 PMC9217132

[45] Stefanovski L , Triebkorn P , Spiegler A , Diaz-Cortes MA , Solodkin A , Jirsa V , McIntosh AR , Ritter P , Alzheimer’s Disease Neuroimaging Initiative (2019) Linking molecular pathways and large-scale computational modeling to assess candidate disease mechanisms and pharmacodynamics in Alzheimer’s disease. Front Comput Neurosci 13, 13–54. 31456676 10.3389/fncom.2019.00054 PMC6700386

[46] Colclough GL , Woolrich MW , Tewarie PK , Brookes MJ , Quinn AJ , Smith SM (2016) How reliable are MEG resting-state connectivity metrics? Neuroimage 138, 284–293. 27262239 10.1016/j.neuroimage.2016.05.070 PMC5056955

[47] O’Neill GC , Barratt EL , Hunt BA , Tewarie PK , Brookes MJ (2015) Measuring electrophysiological connectivity by power envelope correlation: A technical review on MEG methods. Phys Med Biol 60, R271–295. 26447925 10.1088/0031-9155/60/21/R271

[48] Kudo K , Ranasinghe KG , Morise H , Syed F , Sekihara K , Rankin KP , Miller BL , Kramer JH , Rabinovici GD , Vossel K , Kirsch HE , Nagarajan SS (2023) (2023) Neurophysiological trajectories in Alzheimer’s disease progression. bioRxiv, doi :10.1101/2023.05.18.541379 [Preprint]. Posted May 22, 2023. PMC10977971 38546337

[49] Youssef N , Xiao S , Liu M , Lian H , Li R , Chen X , Zhang W , Zheng X , Li Y , Li Y (2021) Functional brain networks in mild cognitive impairment based on resting electroencephalography signals. Front Comput Neurosci 15, 698386. 34776913 10.3389/fncom.2021.698386 PMC8579961

[50] Yu M , Engels MMA , Hillebrand A , van Straaten ECW , Gouw AA , Teunissen C , van der Flier WM , Scheltens P , Stam CJ (2017) Selective impairment of hippocampus and posterior hub areas in Alzheimer’s disease: An MEG-based multiplex network study. Brain 140, 1466–1485. 28334883 10.1093/brain/awx050

[51] Yu M (2020) Benchmarking metrics for inferring functional connectivity from multi-channel EEG and MEG: A simulation study. Chaos 30, 123124. 33380013 10.1063/5.0018826

[52] Vinck M , Oostenveld R , van Wingerden M , Battaglia F , Pennartz CM (2011) An improved index of phase-synchronization for electrophysiological data in the presence of volume-conduction, noise and sample-size bias. Neuroimage 55, 1548–1565. 21276857 10.1016/j.neuroimage.2011.01.055

[53] Waschke L , Kloosterman NA , Obleser J , Garrett DD (2021) Behavior needs neural variability. Neuron 109, 751–766. 33596406 10.1016/j.neuron.2021.01.023

[54] Breakspear M , Williams LM , Stam CJ (2004) A novel method for the topographic analysis of neural activity reveals formation and dissolution of ‘Dynamic Cell Assemblies’. J Comput Neurosci 16, 49–68. 14707544 10.1023/b:jcns.0000004841.66897.7d

[55] Gschwandtner U , Bogaarts G , Roth V , Fuhr P (2023) Prediction of cognitive decline in Parkinson’s disease (PD) patients with electroencephalography (EEG) connectivity characterized by time-between-phase-crossing (TBPC). Sci Rep 13, 5093. 36991083 10.1038/s41598-023-32345-6 PMC10060251

[56] Lee H , Noh GJ , Joo P , Choi BM , Silverstein BH , Kim M , Wang J , Jung WS , Kim S (2017) Diversity of functional connectivity patterns is reduced in propofol-induced unconsciousness. Hum Brain Mapp 38, 4980–4995. 28670685 10.1002/hbm.23708 PMC6866820

[57] Stam CJ , Hillebrand A , Wang H , Van Mieghem P (2010) Emergence of modular structure in a large-scale brain network with interactions between dynamics and connectivity. Front Comput Neurosci 4, 133. 20953245 10.3389/fncom.2010.00133 PMC2955452

