# Supplementary material for: Network Hyperexcitability in Early-Stage Alzheimer’s Disease: Evaluation of Functional Connectivity Biomarkers in a Computational Disease Model
Source: J Alzheimers Dis. 2024 Jun 11;99(4):1333–48. doi: 10.3233/JAD-230825 (PMC11191539; doi:10.3233/JAD-230825)

Supplementary Material

Network Hyperexcitability in Early-Stage Alzheimer's Disease: Evaluation of Functional Connectivity Biomarkers in a Computational Disease Model

Comparison of empirical MEG data with ADD model

As described in the Materials and Methods section in the main text, the Activity dependent degeneration (ADD) algorithm aims to simulate progressive changes in structural and functional networks induced by damage caused by excessive firing of excitatory neurons. The damage to structural networks, changes in spectral features, and changes in excitatory and inhibitory firing rates (and their balance) are shown in Figs. 2-4 in the main text. Here, we present further evidence for the validity of this model by comparing empirical resting-state recordings of MEG (magnetoencephalography) to the ADD model. For this purpose, we re-analyzed a dataset of 18 persons with subjective cognitive decline (SCD), 18 subjects with mild cognitive impairment (MCI), and 18 subjects with Alzheimer's disease (AD). Spectral features of these data were previously described in detail in an earlier paper in this journal [1]. Results with the joint permutation entropy have also been described previously [2]. Descriptive information on these groups can be found in Supplementary Table 1. Further background information can be found in [1,2].

For the present analysis, we first computed spectral features for 20 consecutive epochs (sample frequency: 1250 Hz; length 3.2768 s (4096 samples); 78 AAL ROIs, corresponding to the 78 ROIs used for the ADD model). Since relative power in the theta band, averaged over all 78 ROIs, is a promising biomarker in early AD we used this to match the empirical data to the model. Specifically, for each epoch of each subject we determined the model time step (range: 1-100) where the squared difference between model and empirical average relative theta power obtained the smallest value. With this approach we determined for each subject an average (over 20 epochs) best matching time to the ADD model. Since each model time also corresponds to specific values of underlying model parameters such as excitatory and inhibitory firing rates, E/I balance, coupling strengths between excitatory and inhibitory populations, and between thalamic input and excitatory populations, these we taken into account as well. Finally, we determined whether optimal matching times and internal model parameters were significantly different

between the MCI group compared to the SCD group and the AD group compared to the SCD group.

Results of this analysis are shown in Supplementary Table 2. As can be seen, the SCD group showed an optimal match to the ADD model at an earlier time step (mean 20.01 SD 2.88) compared to the MCI group (mean 23.52; SD 4.25) and the AD group (mean 35.06; SD 16.48). The matching times of the MCI and AD groups were both significantly later than the of the SCD group ($p < 0.005$). This shows that progressive pathology from SCD to MCI to AD corresponds to later matching times (later simulated stages of the degenerative process) in the ADD model. Of interest, this also allows to infer from the model internal features such as firing rates and connection strengths which are not directly accessible in empirical data (columns 2-7 in Supplementary Table 2). These findings suggest significantly increased excitatory and decreased inhibitory firing rates and increased E/I balance in MCI and even more so in AD. This is accompanied by a loss of structural connectivity between the excitatory and inhibitory populations (parameters C1 and C2) and a loss of thalamic input (Pt).

Next, we evaluated whether and to what extent the patterns of chance in functional connectivity measures predicted by the ADD model could be found in empirical MEG recordings of the SCD, MCI, and AD groups. In particular, we were interested in the prediction by the model that new measures (JPE and PLT) would be more sensitive to early pathology (MCI phase as opposed to AD phase) compared to conventional measures (AECc and PLI), in particular in the theta band. Results of this analysis are shown in Supplementary Table 3 and Supplementary Figure 1. Note that the results for the JPE of this dataset have been described in [2]. Significant changes in functional connectivity in the AD group compared to the SCD group could be demonstrated with the AECc in the alpha and beta band, with the PLI in the delta band, with the JPE in the theta band, and with the PLT in the theta and beta band. Significant changes in functional connectivity between the MCI group and the SCD group could only be demonstrated with the JPE and the PLT in the theta band. The empirical data therefore show a pattern of changes, in particular in the theta band, which is qualitatively similar to the predictions made by the model. In particular, the main prediction of the model—superiority of JPE and PLT in the theta band in detecting abnormalities—in the early phase along the AD spectrum is confirmed by the empirical findings.

REFERENCES

[1] Luppi JJ, Schoonhoven DN, van Nifterick AM, Gouw AA, Hillebrand A, Scheltens P, Stam CJ, de Haan W (2022) Oscillatory activity of the hippocampus in prodromal Alzheimer's disease: a source-space magnetoencephalography study. *J Alzheimers Dis* **87**, 317–333.

[2] Scheijbeler EP, van Nifterick AM, Stam CJ, Hillebrand A, Gouw AA, de Haan W (2022) Network-level permutation entropy of resting-state MEG recordings: A novel biomarker for early-stage Alzheimer's disease? *Netw Neurosci* **6**, 382-400.

Supplementary Table 1. Subject characteristics (from Luppi et al., [1])

	SCD	MCI	AD
**n**	18	18	18
**Age (y)**	64.2 (± 6.1)	64.1 (± 6.2)	63.8 (± 6.5)
**M/F (n)**	8/10	9/9	9/9
**Mean MMSE score (points)**	27.8 (± 2.1)	25.8 (± 1.9)	17.9 (± 4.7)

SCD, subjective cognitive decline; MCI, amnestic mild cognitive impairment with positive amyloid-β biomarkers for AD; AD, Alzheimer's disease dementia with positive amyloid-β biomarkers; MMSE, Mini-Mental State Examination; ns, no significant differences. **significance level of $p < 0.001$ (MCI versus SCD and AD versus MCI. Numbers between brackets represent standard deviations.

Supplementary Table 2. Fitting empirical MEG recordings to ADD model.

	Time	E rate	I rate	E/(E+I)	C1	C2	Pt
**SCD**	20.01	40.35	58.93	40.64	25.68	2.41	441.31
**SD**	2.88	0.78	0.81	0.80	0.77	0.07	13.24
**MCI**	23.52**	41.25**	57.92**	41.60**	24.77**	2.32**	425.62**
**SD**	4.25	1.04	1.27	1.17	1.08	0.10	18.63
**AD**	35.06**	42.96**	54.01**	44.60**	22.22**	2.08**	381.84**
**SD**	16.48	2.00	5.84	4.22	3.46	0.33	59.48

Time, number of time steps in ADD model corresponding to optimal fit with empirical data; E rate, firing rate of excitatory population in spikes/second; E rate, firing rate of inhibitory population in spikes per second; E/(E+I), balance between excitatory and inhibitory firing rates; C1, coupling strength between excitatory and inhibitory populations; C2, coupling strength between inhibitory and excitatory populations; Pt, thalamic input to excitatory population in spikes per second; SCD, subjective cognitive decline; MCI, mild cognitive impairment; AD, Alzheimer's disease; SD, standard deviation. **$p<0.005$ (comparison of MCI or AD to SCD; Permutation test using BrainWave).

Supplementary Table 3. Mean values (SD: standard deviation) of functional connectivity measures (AECc, PLI, JPE, and PLT) computed over 20 epochs for SCD (subjective cognitive decline, n=18), MCI (mild cognitive impairment, n=18) and AD (Alzheimer's disease, n=18) groups in the delta, theta, alpha, and beta frequency bands. *p<0.05; **p<0.005.

	Delta band (0.5-4 Hz)				Theta band (4-8 Hz)		
	**SCD**	**MCI**	**AD**		**SCD**	**MCI**	**AD**
AECc	0.09	0.093	0.094	AECc	0.09	0.097	0.092
SD	0.006	0.009	0.014	SD	0.01	0.012	0.008
PLI	0.218	0.216	0.212*	PLI	0.184	0.186	0.186
SD	0.006	0.006	0.006	SD	0.003	0.006	0.004
JPE	0.639	0.633	0.623	JPE	0.529	0.510*	0.506**
SD	0.016	0.015	0.025	SD	0.020	0.018	0.018
PLT	0.130	0.130	0.130	PLT	0.085	0.095*	0.100**
SD	0.005	0.005	0.008	SD	0.008	0.011	0.013

	Alpha band (8-13 Hz)				Beta band (13-30 Hz)		
	**SCD**	**MCI**	**AD**		**SCD**	**MCI**	**AD**
AECc	0.098	0.092	0.084*	AECc	0.067	0.058	0.054**
SD	0.013	0.010	0.009	SD	0.015	0.010	0.004
PLI	0.176	0.175	0.173	PLI	0.098	0.099	0.096
SD	0.011	0.006	0.008	SD	0.003	0.004	0.002
JPE	0.478	0.474	0.476	JPE	0.425	0.425	0.428
SD	0.006	0.003	0.005	SD	0.006	0.004	0.007
PLT	0.086	0.095	0.084	PLT	0.054	0.055	0.051*
SD	0.013	0.011	0.01	SD	0.004	0.003	0.003

Supplementary Figure 1. Changes in mean functional connectivity (averaged over all 78 cortical ROIs of AAL atlas and 20 consecutive epochs) comparing either the MCI (18 subjects, n=18) or the AD (18 subjects, n=18) group to the SCD (subjective cognitive decline, n=18) group. Magnitude of change is expressed as Cohen's effect size *d* (difference in means divided by mean of the two corresponding standard deviations). Results are shown for all four functional connectivity measures investigated (AECc, PLI, JPE, and PLT), and in four frequency bands (delta, theta, alpha, and beta). The corresponding mean values, standard deviations and significances are shown in Supplementary Table 3. Note the large effect sizes of the JPE and PLT compared to the AECc and PLI in the theta band.

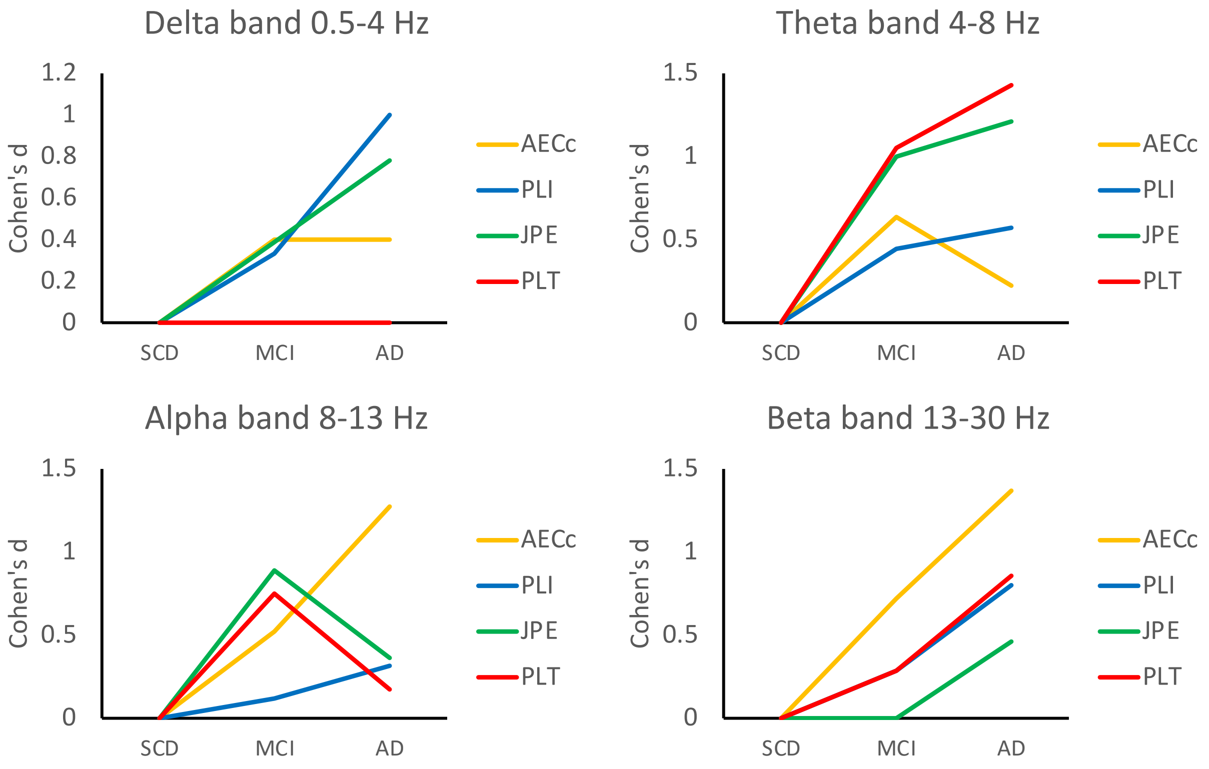

Supplement: Supplementary Material [file jad-99-jad230825-s001.pdf]
